# Optimized Field Collection and Gut Dissection Workflows for Microbiome Studies of the Citrus Root Weevil, *Diaprepes abbreviatus*

**DOI:** 10.21769/BioProtoc.5761

**Published:** 2026-07-20

**Authors:** Paola G. Figueroa-Pratts, Tasha M. Santiago-Rodriguez, Imilce A. Rodriguez-Fernandez

**Affiliations:** 1 Microbiology and Genetics Laboratory, Department of Biology, University of Puerto Rico Rio Piedras, San Juan, Puerto Rico; 2 Environmental Microbiology Laboratory, Department of Biology, University of Puerto Rico Rio Piedras, San Juan, Puerto Rico; 3 The Alkek Center for Metagenomics and Microbiome Research, Department of Molecular Virology and Microbiology, Baylor College of Medicine, Houston, TX, USA; 4 Department of Molecular Virology and Microbiology, Baylor College of Medicine, Houston, TX, USA

**Keywords:** *Diaprepes abbreviatus*, Field collection, Gut anatomy, Insect microbiome, Contamination control

## Abstract

Careful dissection of insect gut tissues is essential for microbiome studies to ensure accurate characterization of internal microbial communities and preservation of DNA integrity. Because insect-associated microbiomes are highly sensitive to contamination, effective removal of external microbes prior to dissection is critical to minimize bias in downstream analyses. While ethanol- and bleach-based surface sterilization methods are commonly used, standardized workflows integrating field collection, sterilization, and dissection remain limited. Here, we present a step-by-step protocol for the field collection, surface sterilization, and dissection of gut tissues from the agricultural pest *Diaprepes abbreviatus* (Coleoptera: Curculionidae), optimized for genomic DNA extraction and microbiome analyses. Using wild-caught specimens, this workflow incorporates a rigorous surface sterilization and dissection strategy that minimizes external contamination while preserving biologically relevant microbial signatures and DNA integrity for downstream microbiome analyses. The protocol provides a standardized framework for insect gut microbiome studies and can be broadly adapted to other wild-caught insect species requiring careful collection, disinfection, and sterile dissection prior to molecular analysis. The protocol integrates field collection and laboratory processing steps into a streamlined workflow that minimizes contamination while preserving tissue integrity for downstream applications.

Key features

• Designed for wild-caught *Diaprepes abbreviatus* collected directly from agricultural host trees, this protocol can also be adapted for other insect species.

• Integrates field collection, surface sterilization, and sterile gut dissection into a single workflow to minimize contamination.

• Sequential ethanol and diluted bleach treatment effectively removes external microbes prior to dissection.

• Enables isolation of intact gut tissues suitable for high-quality DNA extraction and downstream microbiome sequencing.

## Graphical overview


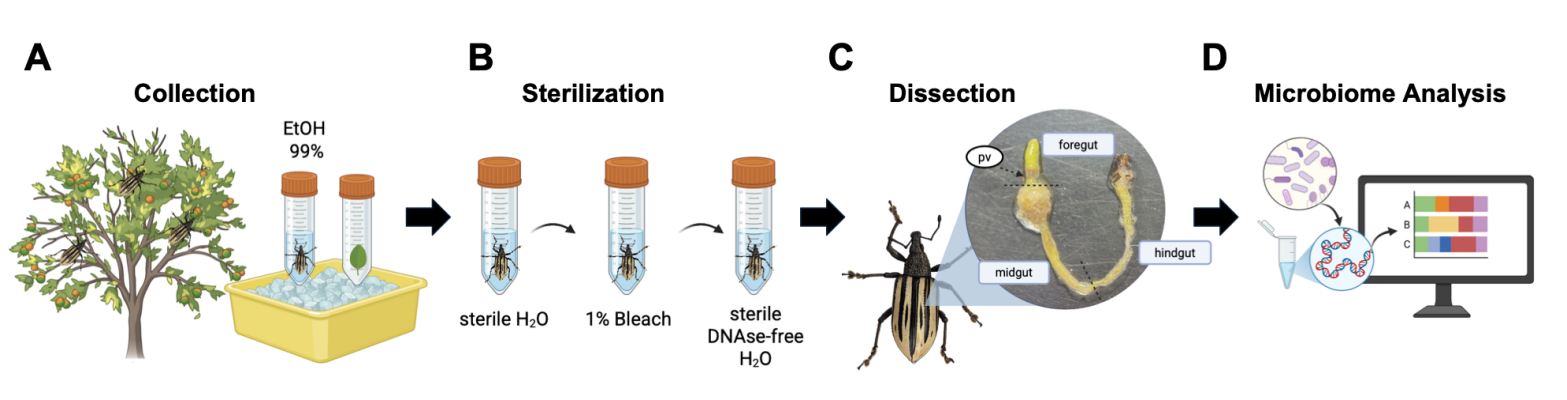


**Protocol overview.** Illustration of the workflow including field collection (A), surface sterilization (B), gut dissection (C), and downstream applications for microbiome studies (D). Figure created using Biorender.

## Background

The citrus root weevil, *Diaprepes abbreviatus*, is an agricultural pest that affects a wide range of plant species and presents significant challenges for crop production in the Caribbean and the United States [1,2]. As an invasive species with resistance to multiple control strategies, there is a growing need to better understand its biology to identify alternative management approaches [3,4]. Insect gut microbiome studies have highlighted the importance of microbial communities in digestion, nutrient acquisition, and host fitness, suggesting that characterization of these communities may provide insights into pest survival and adaptation [5]. Advances in genomic technologies, such as 16S rRNA gene and shotgun metagenomic sequencing, have enabled analysis of insect-associated microbiota. However, the successful use of these technologies and the accuracy of the resulting data rely on proper sample collection, handling, preparation, and dissection.

Previous studies have employed ethanol preservation and bleach-based surface sterilization to minimize external contamination prior to microbiome analyses. While these methods are widely used, there is limited standardization of workflows that integrate field collection, surface sterilization, and gut dissection, particularly for *D. abbreviatus*. Variability in sample handling and sterilization approaches can influence microbial profiles, particularly in low-biomass samples, highlighting the need for reproducible protocols [6,7].

Here, we present a protocol that combines field collection of wild-caught insects, ethanol-based preservation, sequential bleach surface sterilization, and sterile dissection techniques to isolate gut tissues for downstream DNA extraction and microbiome analysis. This integrated workflow minimizes external contamination while preserving internal microbial communities and provides a reproducible framework for insect microbiome studies [8]. A limitation of this protocol is the requirement for sterile handling conditions during dissection, and optimization may be necessary for other insect species with different anatomical characteristics. Nevertheless, this approach can be adapted for use in additional insect systems where accurate characterization of gut-associated microbiota is required.

## Materials and reagents

**Biological materials**

1. Adult *Diaprepes abbreviatus* insects (collected in the field; 18.3722° N, 66.5328° W, Florida, Puerto Rico)

**Reagents**

1. 10× phosphate-buffered saline (PBS) (Fisher Scientific, catalog number: BP399500)

2. Ultrapure H_2_O, sterile, autoclaved

3. Clorox bleach with 3.65% sodium hypochlorite (NaOCl) (The Clorox Company)

4. DNA-free water, sterile (nuclease-free DEPC-treated water) (VWR, catalog number: 10220-382)

5. Ethanol 99% molecular grade (Sigma-Aldrich, catalog number: E7023)

**Solutions**

1. 1× PBS (see Recipes)

2. 1% commercial bleach solution (see Recipes)

**Recipes**

**1. 1× PBS**

**Table:** 

Reagent	Final concentration	Quantity or volume
Ultrapure H_2_O	-	900 mL
PBS	10×	100 mL
Total	1×	1,000 mL

Sterilize using a syringe filter (0.22 μm) and store at room temperature.

**2. 1% commercial bleach solution**

**Table:** 

Reagent	Final concentration	Quantity or volume
Clorox bleach	3.65% NaOCl	68.5 mL
Distilled H_2_O	-	181.5 mL
Total	1%	250 mL

Store at room temperature.

**Laboratory supplies**

1. 50 mL Falcon^®^ centrifuge tubes, polypropylene, sterile (Corning, VWR/Avantor, catalog number: 21008-940)

2. Premium microcentrifuge tubes, 2.0 mL, sterilized by autoclaving (Fisherbrand^TM^, Fisher Scientific^TM^, catalog number: 05-408-138)

3. Premium microcentrifuge tubes, 1.5 mL, sterilized by autoclaving (Fisherbrand^TM^, Fisher Scientific^TM^, catalog number: 05-408-129)

4. Pyrex beaker 600 mL with foil lid, sterile (Corning, Fisher Scientific^TM^, catalog number: 02-540M)

5. Syringe filters, sterile, mixed cellulose ester, 25 mm, 0.20 μm (Basix^TM^, Fisher Scientific^TM^, catalog number: 13-1001-02)

6. Sterile disposable syringe (10 mL) without needle (Nipro Corporation, Osaka Jan, catalog number: 531-8510)

7. Petri dishes with clear lid 100 mm × 15 mm, sterile (Fisherbrand^TM^, Fisher Scientific^TM^, catalog number: FB0875712)

8. Ice

9. Dry ice

10. Alcohol burners

11. Ruler

12. Kimwipes^TM^ Delicate task wipers (Kimberly-Clark Professional, Fisher Scientific^TM^, catalog number: 06-666)

## Equipment

1. Complete 10GSM Medical Student Anatomy Dissection Kit, sterile, autoclaved, containing 4.5-inch dissecting forceps (medium point) and 4.5-inch 1 × 2 teeth tissue forceps (DR Instruments, model: 10GSM) (Amazon Standard Identification Number: B0091N6QF0)

2. Fine Dumont #5 Forceps, Dumostar, biological (Electron Microscopy Sciences, catalog number: 72705-12)

3. Smart Phone, iPhone 14 Pro Max (Apple, model: MQ8V3LL/A) with Weather application by Apple, Inc. to collect environmental conditions

4. Nikon stereomicroscope (zoom: 0.7–4.5×, eyepiece: 10×)

5. Analytical balance

6. Refrigerator (4 °C)

7. Ultra-low freezer (-80 °C)

## Procedure

**A. Field collection of insects**

1. Before field collection, prepare one 50 mL Falcon tube with 40 mL of 99% molecular-grade ethanol for every four insects and place the tubes on ice in a cooler. In addition, bring five 50 mL Falcon tubes without ethanol to collect leaves directly from the trees where the insects are found.

a. If needed, bring a sterile 500 mL beaker with a foil cover to collect more leaves.

b. Aim to collect more insects than needed, as some dissections may not yield usable guts due to the brittleness of gut samples or variability in precision of dissection techniques that can result in inappropriate sample handling.

See Troubleshooting Problem 4 to prevent damage to tissues and preserve viable samples.

*Note: We recommend planning field collection and dissection of insects to take place within 24 h, as storage in 99% ethanol for longer periods can cause tissues to become brittle. Prepare all required materials at least one day in advance to save time, except reagents that need to be freshly prepared for use, such as the 1% NaOCl commercial bleach solution.*

2. Upon arrival at the field, record environmental and climate conditions data such as temperature, humidity, pressure, elevation, and coordinates with a smartphone or handheld device before putting on gloves. The Weather application by Apple, Inc. can be used to collect this data.

3. Wear gloves and avoid touching phones, clothing, or face during sample collection. Collect the insects by hand directly from the trees and place them in a labeled 50 mL Falcon tube containing 40 mL of 99% molecular-grade ethanol. Collect leaves from the same trees where insects were found and place them promptly in 50 mL Falcon tubes without ethanol.

a. Place 4–5 insects per tube. Ensure insects are fully submerged in ethanol.

b. Aim to collect at least 15 leaves from each tree where insects are found to ensure representative environmental sampling.

c. Use a sterile 500 mL beaker if leaves do not fit in Falcon tubes.

*Notes:*

*1. The insects we collected were feeding on lemon trees (*Citrus aurantifolia*) from a local farm in Puerto Rico, located in the municipality of Florida. The insects typically could be found gathering in pairs or scattered throughout the tree, mostly hiding in the upper part of the tree below the leaves, especially those that had lemon flower blossoms nearby. Most of the insects frequented healthy trees with new green leaves. The insects were observed under predominantly sunny conditions, with little to no recent rainfall, and 2–3 weeks after pesticide treatment on the tree farm.*

*2. To minimize sample variability, we collected insects at the same time from the same farm and citrus tree type during all studies. We also ensured that we collected as many insects as possible for each dissection procedure during a single field trip to prevent variability in sample collection dates and weather conditions.*

4. Keep insect samples and leaf samples on ice and transport them directly to the laboratory.

**Pause point:** In the laboratory, insects can be stored in 99% ethanol at 4 °C for up to 24 h to prevent tissue brittleness. Leaves can be kept at room temperature until processing.

*Notes:*

*1. Our leaves were processed approximately 20 days after collection. Since the leaves were hand-collected (with clean gloves) directly from the trees and stored in sterile tubes, the processing of the leaves consisted of weighing the leaves, breaking them into smaller pieces, and pooling them into two sample tubes with approximately 30 leaves per sample. These samples were then packaged and shipped for DNA extraction and sequencing following the standards set by the sequencing company (Zymo Research). For our study, we decided to store the leaves without ethanol as we were interested in microbes found in the surface and inside the leaf tissue. However, we cannot exclude the possibility that longer storage of raw leaf samples (without a preservative) could affect microbial community preservation. We recommend that researchers evaluate whether this parameter is critical for their study objectives and determine whether preserving leaf samples in a stabilization solution such as Zymo DNA/RNA Shield is necessary.*

*2. For our gut microbiome study, we considered the leaf-associated microbial communities as a potential dietary source of microbes detected in the insect gut [9]. Comparing leaf and gut microbiomes allowed us to distinguish environmentally or diet-associated microbes from microbial taxa more likely associated with the gastrointestinal tract [8].*

**See Troubleshooting:** In the metagenomic dataset generated in this study, 20 days elapsed between leaf collection and sequencing. To better preserve microbial communities in future experiments, Zymo DNA/RNA Shield can be used for extended storage. Leaves were weighed after drying prior to sequencing.

5. Record the number of insects and leaves collected. Prepare a data sheet to record sample metadata (e.g., insect ID, collection site, date, sex, and sample type) on the day of dissection (see **Table S1** for an example).

**B. Surface sterilization of insects**

**Figure 1.**
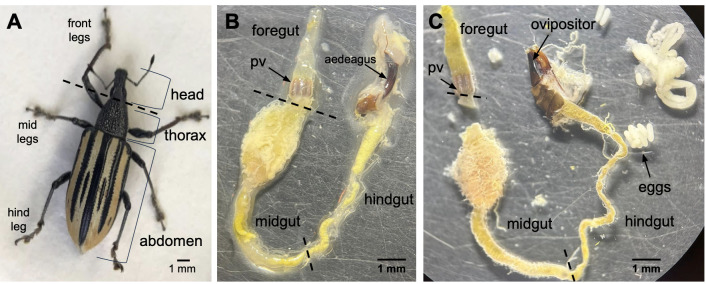
Gut anatomy of adult *Diaprepes abbreviatus.* (A) Adult insect prior to dissection (intact) with relevant anatomical structures indicated. The dashed line indicates the recommended starting dissection site between the head and thorax. (B) Dissected gut of a male showing the foregut, proventriculus (pv), midgut, and hindgut, with exposed male genitalia (aedeagus). (C) Dissected gut of a female showing the foregut, proventriculus (pv), midgut, and hindgut, with exposed female reproductive structures (ovipositor) and eggs. Dashed lines indicate boundaries between foregut, midgut, and hindgut. Scale bars, 1 mm.

1. Before starting, review the insect gut anatomy (**Figure 1**) and work under aseptic conditions, including the use of gloves.

2. Label sterile 2.0 mL microcentrifuge tubes on top and the side with insect ID, collection location, and date of collection. Add 1 mL of sterile 1× PBS to each tube and place on ice. Use one tube per gut. Labeling with a standard numerical order (i.e., 1, 2, 3, …) is recommended to keep track of insects used in each experiment.

3. Prepare the dissection station by spraying surfaces with 70% ethanol. Clean microscope knobs with ethanol and make sure that the work area is disinfected. Disinfect forceps and tweezers by immersing them in 70% ethanol.

4. Remove one insect at a time from the 50 mL Falcon tube containing 99% ethanol using disinfected forceps and gently blot it with a Kimwipe to remove excess ethanol. Using the same forceps, place the insect in the sterile dish on the analytical balance to record its weight. Enter the data into the data sheet generated in step A5 (see Table S1 for an example).

a. You may use the same Petri dish to weigh all insects, as this is done before surface sterilization. Remember to weigh the empty Petri dish beforehand so it can be subtracted from the total weight to accurately record the insect’s weight.

*Note: In the data generated in this study, we used the mean and standard deviation to represent population weight. If individual insect weights are of interest, insects can be separated into new tubes after weighing. However, this will increase resource use during the pre-wash phase.*

**Caution:** 99% ethanol is highly flammable. Do not use an alcohol burner while handling ethanol.

5. Transfer insects (up to four at a time) to an empty sterile 50 mL Falcon tube and add 20 mL of sterile distilled water. Gently invert the tube for 30 s to wash away residual ethanol from the insect surface. Discard the liquid.

6. Repeat the previous step for a total of two washes by gently inverting the tube for 30 s in sterile distilled water during each wash. After the final wash, carefully remove all water while retaining the insects inside the tube. A P1000 pipette can be used to remove the excess liquid while maintaining aseptic techniques.

*Note: The alcohol burner can be used from this point forward, provided appropriate safety precautions are followed.*

**Caution:** Make sure to remove all traces of 99% ethanol using a P1000 pipette before adding bleach (see step B7), as mixing these reagents can generate toxic compounds (e.g., chloroform and acetaldehyde). The tubes can be left open for 2–3 min after washing to allow residual ethanol to evaporate. Dispose of bleach and ethanol separately.

7. Add 20 mL of freshly prepared 1% NaOCl commercial bleach solution (stock at 3.65% sodium hypochlorite) to the tube containing the insects. Shake for approximately 5 s and discard immediately.

*Notes:*

*1. This step serves as a quick pre-wash to prevent dilution of the subsequent bleach treatment by residual water.*

*2. The 1% NaOCl commercial bleach solution was prepared fresh within 24 h of insect collection and dissection, and stored in an opaque, closed container. We recommend preparing this solution fresh for each study and appropriately discarding any unused solution after use.*

8. Add another 20 mL of the 1% NaOCl commercial bleach solution to the tube and shake for 30 s. Gently agitate to ensure all surfaces are exposed. Discard appropriately in polypropylene containers or chemically resistant containers.

**Critical:** Do not exceed 1 min of exposure time to the bleach solution.

*Note: This step is designed to eliminate surface microbes without damaging the insect exoskeleton or internal tissues.*

9. Add 20 mL of sterile DNAse-free water, ensuring the insects are fully submerged in water, and shake for 1 min. Discard the water.

10. Repeat the previous step, discarding the water. The insects are now ready for dissection.

**C. Gut dissection**

1. Add ~1–2 mL of sterile 1× PBS to a new sterile Petri dish and place the surface-sterilized insect in the solution.

2. Disinfect gloves by spraying with 70% ethanol or change gloves before dissection. Avoid touching phones or other non-sterile surfaces during the procedure. If contact occurs, disinfect or replace gloves. Repeat as needed to maintain sterile conditions. Ensure an alcohol burner is available near the work area.

3. Using a sterile scalpel and sterile tweezers, remove the legs from the abdomen, and discard them.

a. The use of 2–3 different sizes of tweezers is recommended. Larger tweezers can be used for handling large tissues and the exoskeleton, while fine tweezers should be used for delicate tissues such as the digestive tract.

*Notes:*

*1. The dissection process takes different durations depending on skill level. For inexperienced users, dissections may require more than 15 min per insect. We recommend practicing dissections with additional insects beforehand. Although we did not observe clear differences in sample quality associated with shorter or longer dissection times, we recommend practicing the procedure before sample collection to minimize dissection time whenever possible and reduce the potential for sample degradation or contamination.*

*2. Before starting the dissection procedure, we recommend reviewing Figure 1 from reference [8], which contains images captured during the dissection process and highlights the different anatomical regions of the insect gut.*

4. Using a scalpel, cut the head at its junction with the thorax (dashed line in Figure 1A) and carefully remove the esophagus or foregut from the head. Keep all dissected gut tissues submerged in sterile PBS at all times.

**Critical:** The foregut is often attached to the head and requires gentle dissection to prevent breakage. Set aside for further analysis if needed.

*Note: The foregut is attached to the proventriculus, which can be used to differentiate tissues during dissection.*

5. Carefully remove the exoskeleton and wings.

**Critical:** The abdomen contains the intestines; therefore, avoid sudden movements when removing the wings, as this may damage the sample. Retain the abdomen in sterile PBS during this step.

6. Remove the abdominal cuticle slowly using fine tweezers. Ensure that intestinal content remains undisturbed throughout the process.

7. Carefully remove surrounding organs and tissues from the gut using tweezers and transfer the gut to the lid of the same sterile Petri dish containing sterile 1× PBS. Ensure that the foregut, midgut, and hindgut remain submerged in sterile PBS.

8. Arrange the dissected tissues and capture an image of the full gut, including associated reproductive organs (if present), for sex determination. Label the image with the insect ID number and include a ruler in the imaging area for scale.

a. For our data, we recorded total gut length in centimeters and performed this step as quickly as possible to minimize exposure time. Gut length can also be measured from images, provided a scale is included.

b. Females often contain eggs, whereas males lack eggs and may have reproductive structures associated with the hindgut.

c. To determine the insect sex, we primarily relied on the identification of the genitalia after dissection. We did not record sex upon field collection because mating behavior was not observed. We preferred this method because *D. abbreviatus* has been noted to have intrasexual mounting behavior between males and females alike [10]; as such, relying on copulating behavior was not a reliable sex determinant in our study.

*Notes:*

*1. If sex cannot be determined, we recommend excluding the sample if sex is a variable of interest. Researchers should also consider potential sex-dependent differences in gut morphology, microbial composition, physiology, or body size when designing microbiome studies.*

*2. If sex is a variable of interest, we recommend the implementation of genetic testing for sex chromosomes.*

9. Transfer the full gut to a labeled sterile 2.0 mL microcentrifuge tube containing 1 mL of sterile 1× PBS. Place the tube on ice.

*Note: We used one gut per tube (one gut per sample), although this can be adjusted depending on experimental design.*

10. Disinfect forceps, tweezers, and dissection tools by immersing them in 70% ethanol between each specimen to prevent cross-contamination.

11. After completing all dissections, carefully remove all PBS from the tubes using a P1000 or P200 pipette, leaving only the gut tissue. Immediately flash-freeze samples on dry ice.

**Critical:** Freeze samples immediately to preserve microbial DNA integrity.

*Note: A nucleic acid stabilization solution (e.g., Zymo DNA/RNA Shield) (between 0.6 and 1 mL or as directed by the manufacturer’s protocol) can be used to preserve samples at room temperature, eliminating the need for immediate freezing.*

12. Store the gut samples at -80 °C until DNA extraction and sequencing. The leaves collected can be stored at room temperature in the same sterile tubes used for collection. Immediately before the processing of the leaves, we recommend recording the weight of the leaves individually to achieve the desired biomass required for effective DNA extraction. This will depend on the technology to be used and its unique standards.

*Note: Our group outsourced DNA extraction with Zymo Research. The company used the ZymoBIOMICS -96 MagBead DNA kit following the manufacturer’s instructions. The sequencing was processed using NovaSeq X. The specifics of DNA extraction and sequencing methodology are available and described in more detail in [8].*

## Validation of protocol

This protocol was used and evaluated in [8] (see Figure 7). Our group expanded upon previous characterization of bacterial communities from fecal samples using 16S rRNA gene sequencing [9] to include bacterial communities from whole guts and gut regions using metagenomic approaches [8].

The initial protocol, designed to study the microbiome of individual gut regions, used a different collection and dissection workflow. Insects were collected directly from trees without the use of gloves and were kept alive until dissection 24 h later. Preparation steps consisted of surface sterilization with 70% ethanol for 30 s, followed by a 10 s wash with sterile 1× PBS. The legs, head, exoskeleton, and wings were then removed, and the body was washed again in 70% ethanol for 30 s, followed by two washes with sterile 1× PBS under agitation prior to dissection of the thorax and abdomen and subsequent separation of gut regions (Supplementary Methods 1.1 in [8]). However, this protocol, which did not include a 1% bleach treatment, resulted in detectable contamination with human skin–associated microbiota. Subsequent curation of the dataset to remove 18 human-associated taxa (e.g., *Cutibacterium acnes, Staphylococcus epidermidis, Micrococcus luteus*) revealed insufficient elimination of contaminants during collection and dissection (compare Supplementary Table 3 with Supplementary Table 4 in [8]).

In contrast, the optimized protocol described here, used for full-gut isolation in metagenomic analyses, significantly improved sample quality and contamination control (Supplementary Table 2 in [8]). Since our group outsourced DNA extraction and sequencing, the ZymoBIOMICS Microbial Community Standard (Zymo Research, Irvine, CA) was used as a positive control for each DNA extraction and library preparation. A blank from the extraction protocol and a blank from the library preparation were used as negative controls to assess cross-contamination. This workflow enabled the recovery of diverse and biologically relevant gut microbiota, including both unique and shared taxa across independent methods, indicating improved sensitivity and reproducibility.

Importantly, samples processed using this protocol did not show detectable contamination from human-associated microbiota, supporting the effectiveness of the collection, surface sterilization, and dissection procedures. The detection of insect-associated taxa such as *Pantoea, Lactococcus, Pseudomonas, Enterococcus*, and *Spiroplasma*, consistent with results obtained using independent methodologies, further validates the accuracy of this approach (Figure 7 in [8]). Overall, this cross-method comparison supports the utility of the protocol as a robust, reproducible, adaptable, and contamination-aware workflow for the characterization of insect gut microbiomes.

A limitation of this validation is that dedicated negative controls specifically designed to assess the effectiveness of the surface sterilization procedure were not included. Future studies may benefit from sequencing final wash solutions or incorporating additional sterilization controls to directly evaluate the removal of external microbial contaminants.

## General notes and troubleshooting

**General notes**

1. This protocol was designed for the study of insect whole-gut samples. For our data, we preferred to use one full gut per sample. Previous approaches in our research group focused on dissecting individual gut regions; however, these methods resulted in low DNA yields upon quantification. Some tissues, such as the foregut, are highly sensitive to dissection techniques and may yield insufficient material for DNA extraction. In addition, the proventriculus, a highly sclerotized (hardened) structure, may be resistant to standard homogenization procedures. If gut regions are of interest, optimization during cutting the guts, such as with microscissors, will be required.

2. This protocol can be adapted to different experimental designs by modifying the dissection and sample preparation steps according to study objectives.

3. Variability in microbial profiles may arise from differences in collection time, host plant, or environmental conditions. Because diet has been shown to influence insect gut microbiota composition, the host plant species can contribute substantially to differences in microbial community structure. In our previous study [9], insects from the same farm fed different host plants, including lemon, guava, and passion fruit leaves, exhibited differences in bacterial relative abundance and diversity, suggesting that diet is an important determinant of gut microbiome composition.

Environmental conditions may also influence insect availability during field collection. In our study, recent rainfall or unfavorable weather conditions resulted in lower numbers of insects in the tree farm and difficult insect collection. Obtaining sufficient numbers of insects is important to ensure that the resulting microbial community profile accurately represents the insect population at the collection site. We also observed that *D. abbreviatus* preferred sunny conditions (~28–30 °C) and was more frequently associated with new green and dry leaves, indicating that collection timing and environmental conditions should be carefully considered before field sampling.

For sample preservation, insects were placed in 99% molecular-grade ethanol immediately after collection, transported to the laboratory on ice, and stored at 4 °C upon arrival. Dissections were performed within 24 h of collection. Following dissection, gut samples were stored at -80 °C, whereas leaf samples remained in the original sterile collection tubes at room temperature for approximately 20 days prior to shipment to Zymo Research for DNA extraction and sequencing. This workflow enabled prompt sample preservation under controlled conditions to minimize degradation and maintain microbial integrity.

**Troubleshooting**

**Problem 1:** No foregut visualized after cutting the head of the insect.

Possible cause: Imprecise or overly forceful incision at the base of the head during dissection.

Solution: Use fine, pointed tweezers to gently access the neck region and carefully extract the foregut from the cavity.

**Problem 2:** Presence of human skin–associated bacterial contaminants.

Possible cause: Incomplete elimination of external contaminants prior to dissection.

Solution: Ensure insects are handled with gloves at all times and change or disinfect gloves between samples. Thoroughly disinfect the work area and strictly follow surface sterilization steps before each dissection.

**Problem 3:** Low bacterial abundance after sequencing analysis.

Possible cause: Insufficient tissue input or low biomass during DNA extraction.

Solution: Increase the number of insects per sample (pooling), particularly when working with small or fragile gut tissues.

**Problem 4:** Damaged or unusable gut samples upon dissection.

Possible causes: Insects were left in 99% ethanol after collection for extended amounts of time (more than 24 h), or forceful dissection techniques.

Solution: Have dissections take place as soon as possible after field collection or store the samples submerged in 99% ethanol at 4 °C for up to 24 h to prevent tissue brittleness. We recommend practicing dissections with multiple specimens to increase precision and technique before the collection of viable samples. Make sure to use fine-point tweezers for sensitive tissues to prevent damage during the procedure.

## Supplementary information

The following supporting information can be downloaded here:

1. Table S1. Metadata recording sheet for insect gut microbiome samples, including collection information, specimen characteristics, and dissection notes.
